# Late-night salivary cortisol may be valuable for assessing treatment response in patients with Cushing’s disease: 12-month, Phase III pasireotide study

**DOI:** 10.1007/s12020-016-0978-6

**Published:** 2016-05-21

**Authors:** James W. Findling, Maria Fleseriu, John Newell-Price, Stephan Petersenn, Rosario Pivonello, Albert Kandra, Alberto M. Pedroncelli, Beverly M. K. Biller

**Affiliations:** 1 Division of Endocrinology, Metabolism, and Clinical Nutrition, Medical College of Wisconsin, W129 N7055 Northfield Drive Suite A-203, Menomonee Falls, Milwaukee, WI 53051 USA; 2 Departments of Medicine and Neurological Surgery, Northwest Pituitary Center, Oregon Health & Science University, Portland, OR USA; 3 The Medical School, University of Sheffield, Sheffield, UK; 4 ENDOC Center for Endocrine Tumors, Hamburg, Germany; 5 Dipartimento di Medicina Clinica e Chirurgia, Endocrinologia e Metabolismo, Università Federico II di Napoli, Naples, Italy; 6 Novartis Pharma AG, Basel, Switzerland; 7 Neuroendocrine Clinical Center, Massachusetts General Hospital, Boston, MA USA

**Keywords:** Pasireotide, Cushing’s disease, Salivary cortisol, Urinary free cortisol

## Abstract

Measuring salivary cortisol is a simple, convenient and accurate technique with potential value in monitoring patients with hypercortisolism. This analysis reports changes in late-night salivary cortisol (LNSC) during a 12-month, multicentre, Phase III study of patients with Cushing’s disease who were randomized to pasireotide 600 or 900 μg sc bid. LNSC assessment was an exploratory objective based on a single, optional measurement at midnight ± 1 h on the same day as one of the 24-h urinary free cortisol (UFC) measurements. Of 162 enrolled patients, baseline LNSC was measured in 93. Sixty-seven patients had levels above the upper limit of normal (ULN); median baseline levels were 19.7 and 20.7 nmol/L in the groups subsequently randomized to 600 μg (*n* = 40) and 900 μg (*n* = 27), respectively. Median LNSC levels decreased from baseline to month 12; median changes in patients who had baseline LNSC > ULN in the 600 and 900 μg groups were −13.4 nmol/L (–52.6 %; *n* = 19) and −11.8 nmol/L (–56.1 %; *n* = 14), respectively. LNSC normalized at months 6 and 12 in 25/67 (37.3 %) and 13/67 (19.4 %) patients, respectively; 10/25 and 8/13 patients also had normalized UFC, and 7/25 and 4/13 had partial UFC control (UFC > ULN and ≥50 % decrease from baseline). There was a moderate correlation (*r* = 0.55) on the log scale between individual patient LNSC and UFC values when all time points were pooled. Pasireotide decreased LNSC levels during 12 months of treatment. Salivary cortisol may be a simple, convenient biomarker for assessing treatment response in patients with Cushing’s disease.

## Introduction

The determination of 24-h urinary free cortisol (UFC) levels is commonly used in the diagnosis [1] and subsequent treatment monitoring of patients with Cushing’s disease [2, 3]. However, there are important limitations to the use of UFC. For example, patients must collect a complete 24-h urine sample, which can be a significant challenge for ambulatory patients [4, 5]. Values may not be reliable in patients with high fluid intake [6]. In addition, an analysis in patients with Cushing’s disease demonstrated high intra-patient variability (~50 %) in 24-h UFC measurements that were collected on 4 days over a 2-week period [7].

In recent years, the measurement of salivary cortisol has become a vital tool in the diagnosis of patients with Cushing’s disease. High concordance has been shown between UFC and late-night salivary cortisol (LNSC) when screening for Cushing’s syndrome [8], and the measurement of LNSC levels has high sensitivity (92–100 %) and specificity (93–100 %) in the diagnosis of the disease [9–13]. As with UFC, the collection of salivary samples is simple, non-invasive and convenient as they can be obtained by the patient at home and without the need for specialized equipment. Cortisol levels in saliva are independent of salivary flow rates; furthermore, salivary cortisol is stable at room temperature for at least 2 weeks and samples can be shipped to a reference laboratory for assessment [14, 15].

To date, no large studies in Cushing’s disease have reported on the value of salivary cortisol as a tool for monitoring medical treatment response [16–18]. The current analysis evaluates changes in LNSC levels during treatment with the multireceptor-targeted [19] somatostatin analogue pasireotide in a 12-month, Phase III study. In this study, pasireotide treatment led to decreases in UFC levels and improvements in the signs and symptoms of Cushing’s disease [20].

## Methods

### Patients

Adult patients (aged ≥18 years) with a confirmed diagnosis of persistent, recurrent or de novo (if not surgical candidates) Cushing’s disease were enrolled. Cushing’s disease was defined by a mean 24-h UFC level (calculated from four samples collected within 2 weeks) that was ≥1.5 times the upper limit of normal (ULN), morning plasma adrenocorticotropic hormone level ≥5 ng/L (≥1.1 nmol/L) and a confirmed pituitary source of Cushing’s syndrome. Full details of the inclusion and exclusion criteria have been reported previously [20].

The study was approved by the independent ethics committee, research ethics board or institutional review board at each centre and complied with the ICH Harmonized Tripartite Guidelines for Good Clinical Practice, the Declaration of Helsinki and local laws. All patients provided written informed consent.

### Study design

This was a randomized, double-blind, multicentre, 12-month, Phase III study (Clinicaltrials.gov: NCT00434148). Following screening and appropriate washout of cortisol-lowering medications, patients were randomized to subcutaneous (sc) pasireotide 600 or 900 μg bid. At month 3, patients with UFC levels ≤2× ULN continued on their randomized dose, double-blind, until month 6. All other patients were unblinded and their dose increased by 300 µg bid until month 6. At month 6, patients could enter an open-label phase to month 12, during which time the dose could be titrated by 300 µg bid up to a maximum of 1200 µg bid. Dose reductions in steps of 300 μg bid for drug-related adverse events (AEs) were permitted throughout the study.

### Objectives and assessments

The primary objective of the study was to assess the efficacy of pasireotide sc (600 or 900 μg bid) as measured by the proportion of patients with UFC ≤ ULN at month 6; the results of the primary objective have been published previously and so will not be reported here [20]. Key secondary objectives were to assess changes in clinical signs and symptoms; these results have also been published previously and so will not be reported here [21].

A pre-specified exploratory objective of the study, which is the focus of this report, was to evaluate LNSC levels during pasireotide treatment. LNSC levels were evaluated based on single, optional measurements taken using the Salivette^®^ Cortisol system at midnight (±1 h) during the same day as one of the 24-h UFC collections at the following time points: baseline, 3, 6 and 12 months of treatment. Patients were provided with an instruction sheet telling them how to collect the saliva sample, which they did before brushing their teeth or ≥30 min after, and ≥30 min after eating or drinking; they were required to keep the sample refrigerated.

LNSC levels were determined using enzyme-linked immunosorbent assay (ELISA; RE52611, IBL-Hamburg GmbH, Germany; normal range 0.83–8.3 nmol/L [derived from 725 healthy subjects]; limit of detection 0.41 nmol/L; intra-assay variability of 3.2–7.6 % at 7.0–80.8 nmol/L; inter-assay variability of 6.2–9.1 % at 5.9–72.8 nmol/L; cross-reactivity with cortisone 3.3 %). UFC levels were measured by three central laboratories (Eurofins Technology Services [Suzhou] Co Ltd, Suzhou, China, which measured all the samples from Chinese patients, and Eurofins Medinet BV, Breda, The Netherlands; CRL Medinet Inc, Lenexa, KS, USA, which measured all other samples) monthly for the first 6 months, then every 3 months thereafter. Levels were determined using high-performance liquid chromatography (Alliance^®^ 2795 High Throughput System, UV Waters 2487, Waters Corp, Milford, MA, USA; normal range 30–145 nmol/24 h; limit of quantification 5 nmol/L; intra-assay variability of 0.9–6.1 % at 5–2000 nmol/L; inter-assay precision of 2.4–5.7 % at 15–2000 nmol/L).

### Statistical methods

Only patients with available LNSC measurements at baseline were included in this analysis; these patients were stratified according to whether baseline LNSC was ≤ULN or >ULN. UFC response at month 6 was defined based on levels at month 6: control, UFC levels ≤ULN; partial control, UFC levels >ULN and ≥50 % reduction from baseline; uncontrolled UFC, levels >ULN and <50 % reduction from baseline. LNSC response at month 6 was also defined based on levels at month 6: response, LNSC levels ≤ULN (i.e. normalized); non-response, LNSC levels >ULN. If either UFC or LNSC values at month 6 were missing, they were imputed based on the last available measurement between months 3 and 6 inclusive. Change from baseline in LNSC was initially calculated within each patient, then the overall median change was calculated based on these data.

The correlation between LNSC and UFC was evaluated using Spearman’s rank correlation. Only patients with both UFC and LNSC assessments within the same 24-h period were included at each time point (i.e. baseline, months 3, 6, 9 and 12).

## Results

### Patients with available LNSC measurements at baseline

#### Baseline characteristics

Of the 162 patients enrolled into the Phase III study [20], baseline LNSC levels were available in 93 patients (6- and 12-month data were available from 62 and 45 patients, respectively); of these, there were 15 de novo patients and 78 with persistent/recurrent disease. Forty-eight were randomized to pasireotide 600 μg bid and 45 to pasireotide 900 μg bid (Table 1); median baseline LNSC levels in the two dose groups were 17.3 and 10.3 nmol/L, respectively.

**Table 1:** Patient demographics and characteristics at baseline in 93 patients with LNSC measurements available at baseline

Demographic variable	Pasireotide 600 µg bid (*n* = 48)	Pasireotide 900 µg bid (*n* = 45)
Median age (years)	38.0	37.0
Male:female (*n*)	13:35	9:36
Race [*n* (%)]		
Caucasian	34 (70.8)	30 (66.7)
Black	0 (0)	1 (2.2)
Asian	10 (20.8)	10 (22.2)
Other	4 (8.4)	4 (8.8)
Median time since diagnosis (months)	20.2	38.8
Previous surgery [*n* (%)]	35 (72.9)	37 (82.2)
Median LNSC level (nmol/L)	17.3	10.3

#### Effect of pasireotide on LNSC levels in the group overall (*n* = 93)

LNSC levels decreased overall by a median of 3.6 nmol/L (–31.9 %; *n* = 62) after 6 months of pasireotide treatment; decreases in the 600 and 900 μg groups were 4.9 nmol/L (–26.5 %; *n* = 34) and 2.4 nmol/L (–41.8 %; *n* = 28), respectively (Table 2). The overall median decrease in LNSC after 12 months of treatment was 5.3 nmol/L (–29.2 %; *n* = 45); the decreases in the 600 and 900 μg groups were 7.2 nmol/L (–42.2 %; *n* = 24) and 1.6 nmol/L (–26.1 %; *n* = 21), respectively. Overall mean pasireotide dose increased markedly in the 600 μg group from baseline to month 9, then remained stable to month 12 (Table 2); by month 12, mean daily dose was similar in the two treatment groups.

**Table 2:** Median LNSC and mean pasireotide dose during treatment in the 93 patients with available LNSC measurements at baseline

		Pasireotide 600 µg bid (*n* = 48)	Pasireotide 900 µg bid (*n* = 45)	Overall (*n* = 93)
Baseline	Median LNSC [range (nmol/L)]	17.3 (1.7–552.7)	10.3 (1.4–549.5)	14.4 (1.4–552.7)
Month 3	*n*	40	38	78
	Median LNSC [range (nmol/L)]	11.0 (0–132.2)	8.5 (0.8–82.2)	9.5 (0–132.2)
	Absolute change from baseline	–4.4	–1.9	–3.1
	Percentage change from baseline	–28.1	–28.7	–28.3
	Mean dose ± SD [µg/day]	1133 ± 209	1705 ± 222	1412 ± 359
Month 6	*n*	34	28	62
	Median LNSC [range (nmol/L)]	7.8 (0–71.2)	6.9 (0.8–42.3)	7.5 (0–71.2)
	Absolute change from baseline	–4.9	–2.4	–3.6
	Percentage change from baseline	–26.5	–41.8	–31.9
	Mean dose ± SD [µg/day]	1394 ± 410	1821 ± 382	1587 ± 449
Month 9	*n*	26	25	51
	Median LNSC [range (nmol/L)]	13.0 (1.4–33.8)	8.7 (0.6–408.3)	11.0 (0.6–408.3)
	Absolute change from baseline	–7.5	–1.1	–2.2
	Percentage change from baseline	–24.8	–19.7	–22.6
	Mean dose ± SD [µg/day]	1627 ± 538	1776 ± 474	1700 ± 508
Month 12	*n*	24	21	45
	Median LNSC [range (nmol/L)]	10.2 (1.4–37.2)	8.4 (1.9–57.4)	8.8 (1.4–57.4)
	Absolute change from baseline	–7.2	–1.6	–5.3
	Percentage change from baseline	–42.2	–26.1	–29.2
	Mean dose ± SD [µg/day]	1675 ± 586	1771 ± 483	1720 ± 537

### Patients with baseline LNSC levels ≤ULN or >ULN

Of the 93 patients, 26 (28.0 %) had normal LNSC levels at baseline (median level of 5.7 nmol/L; range 1.0–8.0). Sixty-seven patients (72.0 %) had baseline LNSC levels >ULN: 40 in the pasireotide 600 μg bid group and 27 in the pasireotide 900 μg bid group.

#### Effect of pasireotide on LNSC levels in the group with elevated baseline LNSC (*n* = 67)

**Fig. 1:**
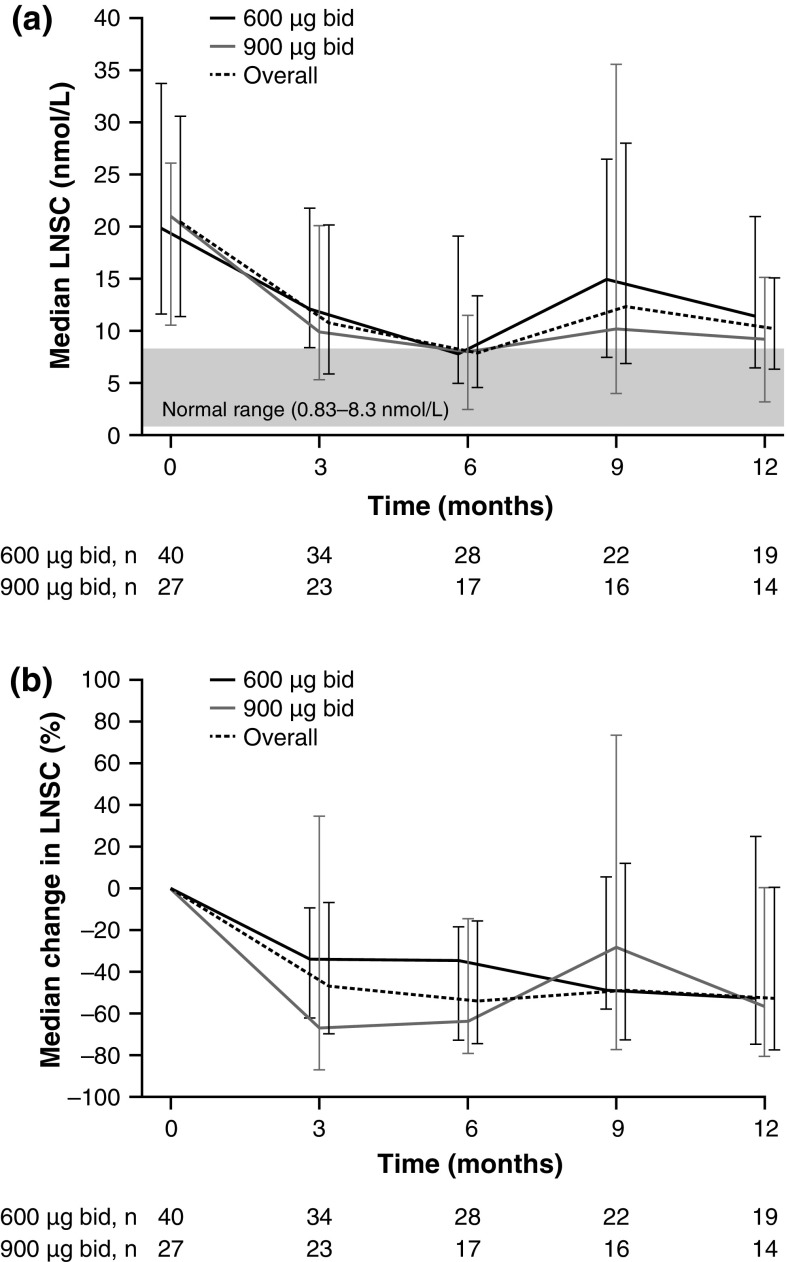
**a** Median absolute LNSC levels (±interquartile range) and **b** median change from baseline in LNSC levels (±interquartile range) in 67 patients with baseline levels >ULN, by randomized dose group and overall. Figure shows patients with available LNSC data at each time point

In patients who had baseline LNSC levels >ULN, median levels decreased from baseline to month 12 during pasireotide treatment (Fig. 1). After 3 months, median LNSC change was −8.4 nmol/L (–46.6 %; *n* = 57) overall, and −5.9 nmol/L (–33.6 %; *n* = 34) and −12.6 nmol/L (–66.7 %; *n* = 23) in the 600 and 900 μg groups, respectively; the equivalent changes after 6 months were −8.1 nmol/L (–53.6 %; *n* = 45), −6.8 nmol/L (–34.2 %; *n* = 28) and −12.1 nmol/L (–63.8 %; *n* = 17). Overall change after 12 months was −11.9 nmol/L (–52.6 %; *n* = 33); changes in the 600 and 900 μg groups were −13.4 nmol/L (–52.6 %; *n* = 14) and −11.8 nmol/L (–56.1 %; *n* = 19), respectively.

#### Effect of pasireotide on LNSC levels in the group with normal baseline LNSC (*n* = 26)

The median changes in patients with baseline LNSC ≤ULN were +0.6 nmol/L (+8.1 %; *n* = 17), +1.4 nmol/L (+23.4 %; *n* = 6) and +0.6 nmol/L (+8.1 %; *n* = 11) at month 6, and +2.3 nmol/L (+42.7 %; *n* = 12), +1.9 nmol/L (+70.4 %; *n* = 5) and +2.6 nmol/L (+37.8 %; *n* = 7) at month 12.

### Normalization of LNSC in patients with baseline levels >ULN

**Fig. 2:**
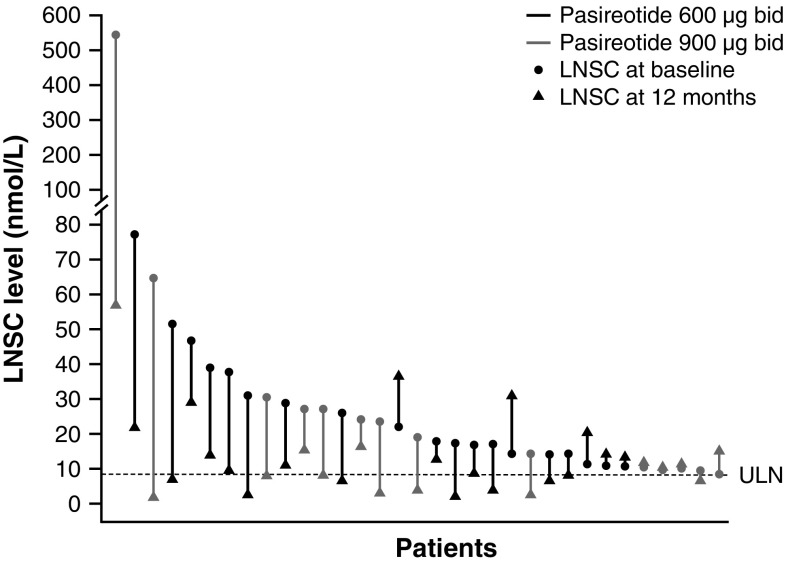
Change in LNSC levels from baseline to month 12 in individual patients with baseline levels >ULN and available month 12 measurements. LNSC measurements were only available in 33/67 patients at month 12

By month 6, LNSC levels had normalized in 25 of the 67 patients who had baseline LNSC > ULN (37.3 %). Ten of the 25 patients with normalized LNSC also had UFC control, while seven patients had partial UFC control; the remaining eight patients had uncontrolled UFC. At month 12, levels had normalized in 13 of the 67 patients who had baseline LNSC > ULN (19.4 %; Fig. 2). Of these 13 patients, eight and four also had UFC control and partial control, respectively; one patient had uncontrolled UFC. Thirty-four patients did not have normalized LNSC levels at month 6 (the remaining eight patients had missing values at months 3 and 6). Five of the 34 patients had UFC control, while eight patients had partial UFC control; the remaining 21 patients had uncontrolled UFC. At month 12, 20 patients did not have normalized LNSC levels (the remaining 34 patients had missing values). Of these 20 patients, five and six also had UFC control and partial control, respectively; nine had uncontrolled UFC.

In both dose groups, median LNSC levels had decreased at 12 months in patients with controlled (–46.8 % in 600 µg group, *n* = 5; −29.4 % in 900 µg group, *n* = 14) and partially controlled (–71.6 % in 600 µg group, *n* = 9; −81.4 % in 900 µg group, *n* = 2) UFC, and increased in uncontrolled patients (+48.9 % in 600 µg group, *n* = 10; +33.1 % in 900 µg group, *n* = 5).

### Correlations: LNSC and UFC

**Fig. 3:**
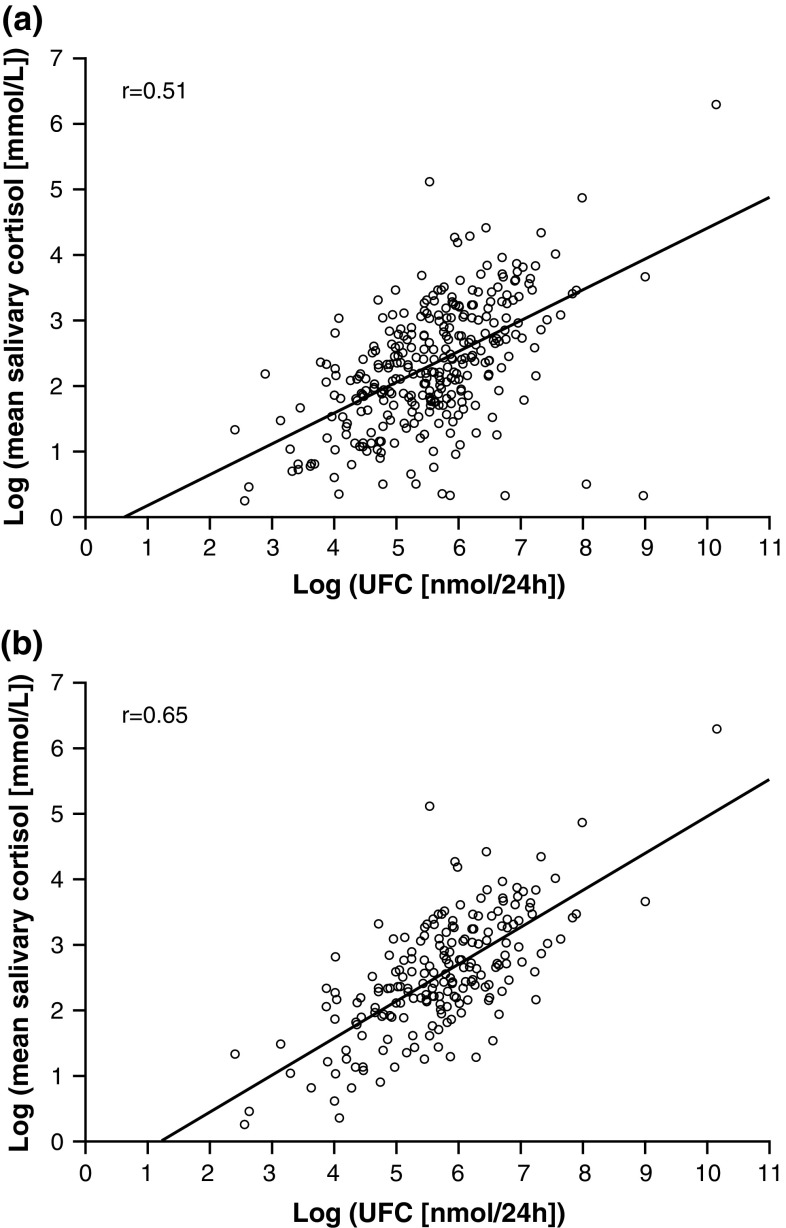
Scatter plot of log LNSC against log UFC at all time points up to month 12 in **a** all patients and **b** patients with baseline LNSC > ULN

The Spearman’s rank correlation between LNSC and UFC was *r* = 0.45 at baseline. Following 6 and 12 months of pasireotide treatment, the correlation was *r* = 0.57 and *r* = 0.33, respectively. When all time points were pooled for all patients, the Spearman’s rank correlation was *r* = 0.51 (Fig. 3a); this was *r* = 0.65 when restricted to only patients with baseline LNSC > ULN (Fig. 3b).

When assessed based on absolute changes in LNSC and UFC during pasireotide treatment, the Spearman’s rank correlation was *r* = 0.24 and *r* = 0.58 at 6 and 12 months, respectively. When all time points were pooled for all patients, the Spearman’s rank correlation was *r* = 0.41.

The correlation between baseline LNSC and percentage change in LNSC for patients who had baseline LNSC levels >ULN was also assessed. The Spearman’s rank correlation was *r* = −0.369 at month 6 and *r* = −0.654 at month 12, whilst the correlation when all time points were pooled was *r* = −0.418.

## Discussion

This exploratory analysis from a subset of patients enrolled in a large Phase III study demonstrated that 12 months of pasireotide treatment led to an overall decrease in LNSC in patients with baseline levels >ULN. In these patients, LNSC levels had normalized in 35.8 % (*n* = 24/67) after 6 months of pasireotide treatment and in 39.4 % (*n* = 13/33) after 12 months. Although the value of LNSC in the diagnosis of Cushing’s disease is known, there is a paucity of data regarding its use as a tool for monitoring medical treatment response. Indeed, there is a general lack of guidance regarding the appropriate method for monitoring treatment of Cushing’s disease, although most available studies have measured changes in UFC levels.

Previous assessment of the effect of medical therapy on LNSC levels is limited to small studies of short duration and the outcomes have been mixed [16–18]. In one analysis of seven patients with elevated baseline LNSC (which correlated significantly with baseline UFC levels; *r* = 0.97, *P* = 0.0002), LNSC levels were reduced in 6/7 patients and UFC levels were decreased in all seven patients after 15 days of pasireotide treatment [17]. A prospective analysis of 14 patients who received cabergoline and ketoconazole combination therapy found that LNSC levels decreased non-significantly from baseline; levels remained above normal in 10/14 patients, even in those with normalized UFC [16]. Finally, in a study of patients who received stepwise medical treatment with pasireotide, cabergoline and ketoconazole, recovery of cortisol diurnal rhythm (CDR; defined by midnight serum and salivary cortisol levels of <75 % of the 09:00 value) was achieved after 80 days in 6/12 patients (1 receiving monotherapy, 1 combination therapy and 4 triple therapy) with disturbed CDR at baseline. CDR did not recover in the six remaining patients, despite the normalization of UFC in five of them [18].

In the present study, the observed decrease in LNSC mirrored the decrease in UFC levels that was noted in the overall population; notably, LNSC levels decreased rapidly (median decrease of 47 % by month 3 in patients with elevated baseline LNSC), similar to the response observed with UFC in the primary analysis [20]. Most of the patients with normalized LNSC also achieved control or partial control of UFC (17/25 [68.0 %] and 12/13 [92.3 %] at months 6 and 12, respectively), which implies an association between changes in LNSC and UFC levels. This was supported by the correlation analysis, which suggested a moderately good correlation (*r* = 0.55) between LNSC and UFC throughout the pasireotide treatment period. Overall, these data suggest that the measurement of LNSC may have value when monitoring medical treatment response in patients with Cushing’s disease. Notably, a number of studies have demonstrated that the assessment of LNSC is an accurate and superior approach to UFC for detecting surgical failure/recurrence of Cushing’s disease [22–26]. In one study, LNSC had 100 % sensitivity in detecting treatment failure, compared with 71 % for UFC [22]. The authors commented that LNSC measurement may detect subtle changes in the dynamics of cortisol secretion that might be missed by a broader evaluation of cortisol secretion using UFC. Indeed, elevated LNSC levels can be observed earlier (mean time of 38.2 months) than elevations in UFC (mean time of 50.6 months) in patients with recurrent Cushing’s disease [27].

Salivary cortisol is usually measured either by an immunoassay (including radioimmunoassay, enzyme-linked immunosorbent assay and electrochemiluminescent immunoassay) or by liquid chromatography–tandem mass spectrometry (LC–MS/MS). Irrespective of the assay used, because of assay variability, it is important that each laboratory develops its own specific reference range for LNSC measurements. Immunoassays are easy to perform and less expensive than LC–MS/MS [28]. In future, the use of automated immunoassays may allow for wider use of LNSC as a diagnostic tool for Cushing’s disease [25] and may help increase its utility as a tool for monitoring treatment. However, the immunoassays inevitably have some cross-reactivity with other corticosteroids, such as cortisone. The salivary glands express corticosteroid 11β-dehydrogenase isozyme 2 (11β-HSD2), which converts cortisol into biologically inactive cortisone; in fact, salivary cortisone levels are 2- to 3-fold greater than salivary cortisol levels. Despite the greater analytical specificity of LC–MS/MS, we do not believe that it has clinical superiority [29, 30] and immunoassays may actually have better diagnostic sensitivity for the diagnosis of Cushing’s syndrome [31, 32]. Similar to UFC, salivary cortisol concentrations can be influenced by a variety of extrinsic factors such as food, exercise, smoking and various emotional/physical disturbances [33–35]. There is substantial day-to-day variation in normal subjects, as well as patients with Cushing’s syndrome [33–35]. Accordingly, current guidelines and longitudinal clinical studies suggest that 2–4 measurements of LNSC should be obtained on different days to confirm the presence or absence of endogenous hypercortisolism.

A major limitation of this study was that because the assessment of LNSC was an exploratory objective, levels were only available from 57.4 % (93/162) of the overall study population and, of those 93, only 45 had LNSC assessments at 12 months. In addition, although patients were instructed to take the salivary cortisol measurements at ‘midnight ± 1 h’, no specific information was noted about when each patient collected their salivary cortisol sample in relation to the time they went to sleep. As the study inclusion criteria were based on elevated mean UFC (1.5× ULN) rather than LNSC levels, 67/93 patients whose LNSC levels were measured had values >ULN at baseline. Similarly, dose adjustments during the study were made based on UFC rather than LNSC levels, which could potentially create a bias. There was an imbalance in baseline LNSC levels between the pasireotide 600 and 900 μg groups (17.3 and 10.3 nmol/L, respectively), which reflects the imbalance seen with UFC in the primary analysis [20]. Ideally, patients should have been stratified for baseline UFC during the randomization process, which may have prevented the observed imbalance in UFC and, subsequently, LNSC across dose groups. The relatively poor sensitivity of LNSC in our study may reflect the fact that only one sample was collected at that time. In addition, the central assay used had a relatively high ULN (8.3 nmol/L), which might be accounted for by cross-reactivity of the salivary cortisol assay with cortisone (3.3 %); it may have been preferable to use a validated diagnostic cut-off for LNSC levels. Finally, because of the known day-to-day variations in salivary cortisol measurements [33–35], it would have been preferable for more than one LNSC measurement to have been taken at baseline and each of the three monthly time points. The Endocrine Society clinical practice guidelines recommend that at least two LNSC measurements be made during the diagnosis of Cushing’s disease [1].

The measurement of LNSC may be a simple, convenient biomarker in Cushing’s disease. The results of this exploratory analysis suggest that LNSC may have value in monitoring medical treatment response in patients with Cushing’s disease. Prospective studies evaluating LNSC levels during medical therapy for Cushing’s disease are warranted.
